# Serum Amyloid P and IgG Exhibit Differential Capabilities in the Activation of the Innate Immune System in Response to Bacillus anthracis Peptidoglycan

**DOI:** 10.1128/IAI.00076-18

**Published:** 2018-04-23

**Authors:** Alanson W. Girton, Narcis I. Popescu, Ravi S. Keshari, Tarea Burgett, Florea Lupu, K. Mark Coggeshall

**Affiliations:** a Department of Microbiology and Immunology, University of Oklahoma Health Sciences Center, Oklahoma City, Oklahoma, USA; b Arthritis and Clinical Immunology, Oklahoma Medical Research Foundation, Oklahoma City, Oklahoma, USA; c Cardiovascular Biology, Oklahoma Medical Research Foundation, Oklahoma City, Oklahoma, USA; University of Illinois at Chicago

**Keywords:** Bacillus anthracis, host response, inflammation, opsonin, peptidoglycan, serum amyloid P

## Abstract

We showed that human IgG supported the response by human innate immune cells to peptidoglycan (PGN) from Bacillus anthracis and PGN-induced complement activation. However, other serum constituents have been shown to interact with peptidoglycan, including the IgG-like soluble pattern recognition receptor serum amyloid P (SAP). Here, we compared the abilities of SAP and of IgG to support monocyte and complement responses to PGN. Utilizing *in vitro* methods, we demonstrate that SAP is superior to IgG in supporting monocyte production of cytokines in response to PGN. Like IgG, the response supported by SAP was enhanced by phagocytosis and signaling kinases, such as Syk, Src, and phosphatidylinositol 3-kinase, that are involved in various cellular processes, including Fc receptor signaling. Unlike IgG, SAP had no effect on the activation of complement in response to PGN. These data demonstrate an opsonophagocytic role for SAP in response to PGN that propagates a cellular response without propagating the formation of the terminal complement complex.

## INTRODUCTION

Infection by Bacillus anthracis via the inhalation route shows a high rate of mortality in humans. We proposed that the mortality in these cases is due to sepsis (1), given the attending bacteremia (2) and signs of inflammation (3, 4) in the terminal stages of infection. The pathophysiology of sepsis by any infectious agent is propagated by pathogen-associated molecular patterns (PAMPs), which are components of pathogens that are recognized by host pattern recognition receptors (PRRs). In sepsis, the large amount of PAMPs in the blood triggers an amplified and dysregulated response by the host's innate immune system. In the absence of effective medical intervention, the consequences of such a massive response are a cytokine storm, disseminated intravascular coagulation, complement activation, immune suppression, organ failure, and eventually death. Peptidoglycan (PGN) triggers sepsis pathophysiology following *in vivo* challenges (5, 6). However, the mechanisms by which PGN induces this damaging pathophysiology are not understood.

PGN is composed of a disaccharide backbone that is highly cross-linked by a short stem peptide. In B. anthracis, the stem peptide is composed of l-alanine, d-glutamic acid, diaminopimelic acid (DAP), and d-alanine. Most Gram-positive bacteria substitute lysine for DAP in the stem peptide. Our studies on human responses to PGN indicate a model in which PGN is opsonized by human serum factors to allow binding and uptake by Fcγ receptors (FcγRs) on innate immune cells (7, 8). Following uptake, PGN is digested in phagolysosomes to monomeric components that stimulate cytoplasmic receptors NOD1 and NOD2 (7). We showed that human IgG can act as a serum opsonin, binding PGN and permitting uptake by Fcγ receptors (9).

Recently, serum amyloid P (SAP) was shown to bind to PGN of the lysine type and support phagocytosis of Staphylococcus aureus whole bacteria by neutrophils via Fcγ receptors (10). SAP is a member of the pentraxin family of proteins, which also includes the acute-phase proteins C-reactive protein (CRP) and pentraxin-3. Unlike the other pentraxins, SAP is constitutively present in human serum at concentrations of between 30 and 50 μg/ml. Similar to IgG, SAP has been demonstrated to interact with components of the complement pathway and with Fcγ receptors (11–16).

In this study, we investigated the relative contributions of SAP and IgG in supporting the innate immune responses to DAP-containing B. anthracis-derived PGN. Utilizing *in vitro* assays, we found that SAP was superior to IgG in supporting monocyte cytokine production in response to PGN while IgG was superior to SAP in supporting complement activation. These findings shed light on the role of human opsonins in mediating immune responses to PAMPs such as PGN during septicemia.

## RESULTS

### The anti-PGN titer is not limiting in monocyte cytokine responses to PGN.

Our previous studies had demonstrated that IgG was capable of supporting monocyte and neutrophil proinflammatory responses to PGN, and all tested human sera contained anti-PGN antibodies, albeit at different titers (9). We therefore hypothesized that if IgG is the sole supporter of monocyte activation in response to PGN, then the extent of the cytokine response should correlate with the titer of anti-PGN IgG.

**FIG 1:**
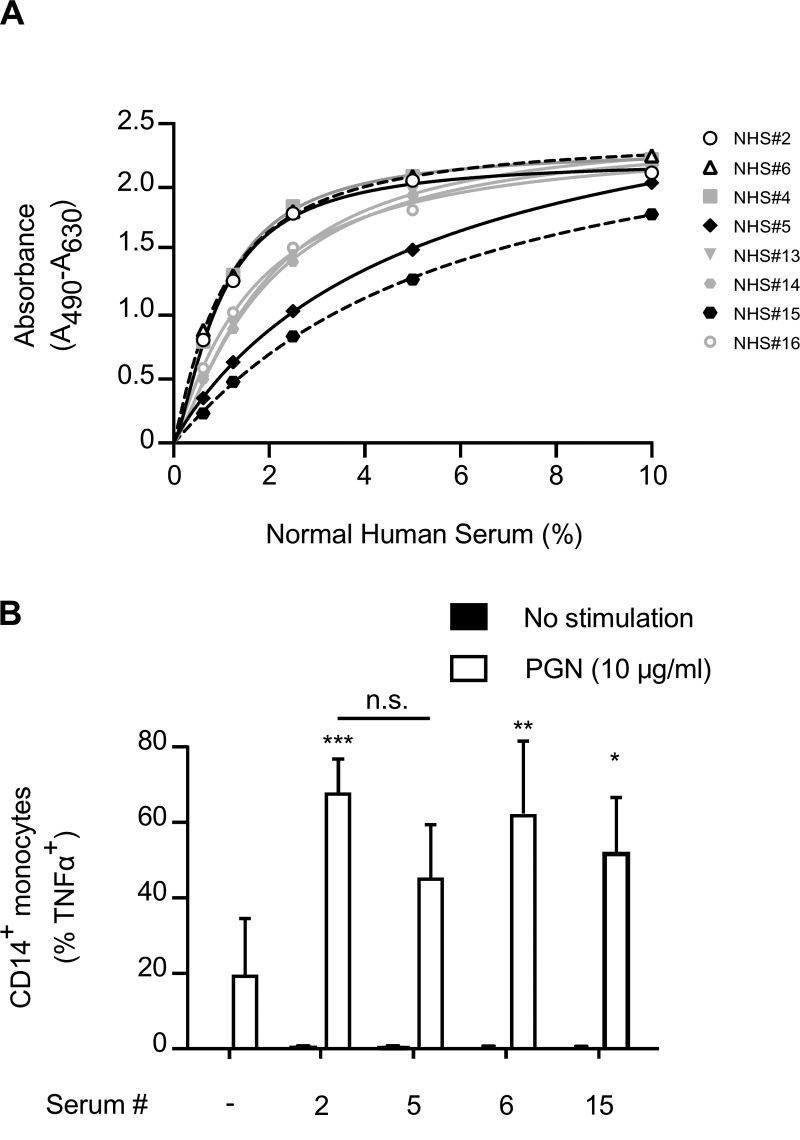
Monocyte responses to PGN are not influenced by IgG titer. (A) Sera from 8 individual donors were analyzed for IgG titers as described in Materials and Methods. From these data we identified 2 high-titer donors (open shapes) and 2 low-titer donors (black-filled shapes). Data shown represent means ± standard errors of the means (SEM) from sera tested in quintuplicate at each concentration. (B) Paired PBMCs were stimulated with PGN in the presence of serum from either the high- or low-titer donors. Monocyte responses to PGN were assayed by intracellular staining for TNF-α. Titration curve was conducted in quintuplicate. Data shown represent means ± standard deviations (SD) from 3 separate donors. Unless connected by a line, significance shown is that compared to cells stimulated with PGN in the presence of FCS only. *, *P* < 0.05; **, *P* < 0.01; ***, *P* < 0.001; n.s., not significant. Two-way ANOVA with Bonferroni posttest was used.

To test our hypothesis, we first determined titers for donors via enzyme-linked immunosorbent assay (ELISA) using PGN-coated wells for capturing anti-PGN antibodies and measured the absorbance at various dilutions of human serum. By using a direct ELISA for anti-PGN activity, we identified two donors containing high anti-PGN IgG titers (NHS 2 and 6) and two donors containing low titers (NHS 5 and 15) (Fig. 1A). Paired peripheral blood mononuclear cell (PBMC) samples were stimulated with PGN in the presence of the four sera. Production of tumor necrosis factor alpha (TNF-α) by CD14^+^ monocytes was measured using intracellular staining and flow cytometry (Fig. 1B). Although the presence of the different human sera supported the TNF-α response, we observed no difference in TNF-α production by CD14^+^ monocytes despite the difference in anti-PGN titers. These data suggest that while IgG is able to support the TNF-α response, IgG is not the sole opsonin in human serum able to do so.

### SAP binds to purified B. anthracis PGN in a calcium-dependent manner and enhances monocyte TNF-α production in response to PGN.

**FIG 2:**
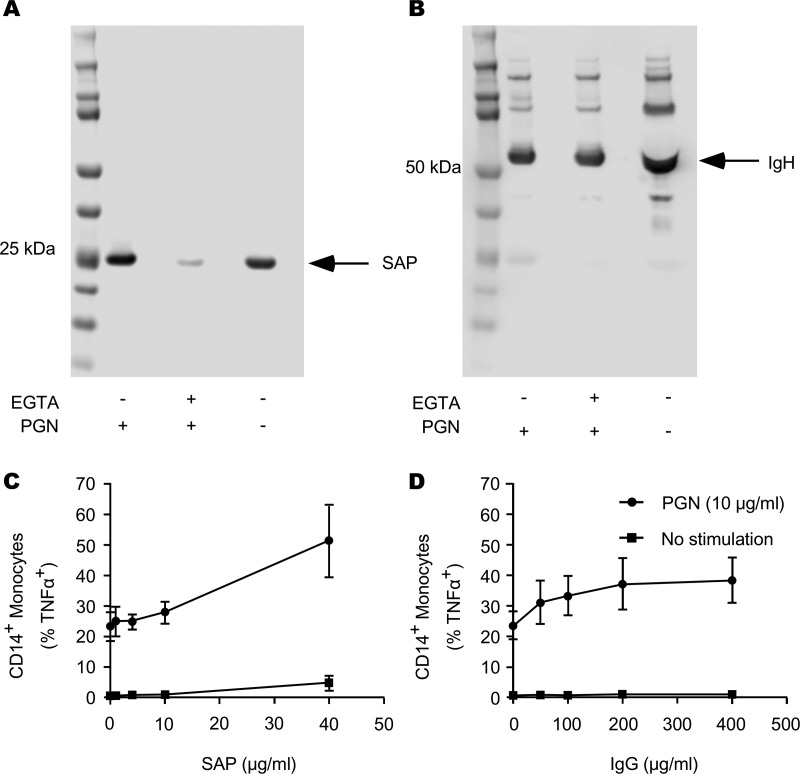
SAP binds B. anthracis PGN in a calcium-dependent manner and enhances monocyte TNF-α production in response to PGN. Human serum was pretreated with EGTA or not pretreated and then incubated with PGN for 1 h at 4°C. PGN was then pelleted by centrifugation and washed. Bound proteins were eluted in LDS sample buffer by heating. (A) Samples were then subjected to PAGE and immunoblotting for SAP. (B) Membranes were then stripped and analyzed for IgG via Western blotting. Images are representative of 3 human donor sera. Subsequently, PBMCs were left unstimulated or were stimulated with PGN (10 μg/ml) in the presence of 1% FCS or 1% FCS supplemented with recombinant SAP (1, 5, 10, or 40 μg/ml) (C) or IgG (50, 100, 200, or 400 μg/ml) (D) for 12 h. Cells were then harvested and stained for CD14 and TNF-α. Flow cytometry was utilized to assess the percentage of CD14 cells that produced TNF-α. Graphs show means ± SD from 3 independent donors.

An earlier study established that SAP was able to opsonize the lysine-type PGN (Lys-type PGN) present in Staphylococcus aureus (10). SAP interaction with the S. aureus Lys-type PGN and subsequent recognition by Fcγ receptors support neutrophil binding and phagocytosis of live S. aureus (10). To assess the ability of SAP to bind to the DAP-type B. anthracis PGN, we incubated human serum from healthy donors with PGN (100 μg), washed away nonbinding proteins, and subsequently immunoblotted for eluted SAP or IgG. We observed that, indeed, SAP was capable of binding B. anthracis PGN, and this binding was dependent on calcium (Fig. 2A). As a control, we observed no effect of EGTA on the binding of IgG to PGN (Fig. 2B). These data show that SAP binds the DAP-type B. anthracis PGN in a calcium-dependent manner, as it does the Lys-type S. aureus-derived PGN. We also tested the ability of SAP and of IgG to support monocyte TNF-α production. We found that both proteins were able to support TNF-α responses in monocytes (Fig. 2C and D). We observed a maximal response for SAP-opsonized PGN at 40 μg/ml SAP, and we observed no saturation within the tested concentration range. The IgG-supported response was maximal and saturable at 200 μg/ml. All tested concentrations are within circulating blood concentrations in humans and are thus physiologically relevant.

### SAP is superior to IgG in supporting monocyte activation in response to PGN.

**FIG 3:**
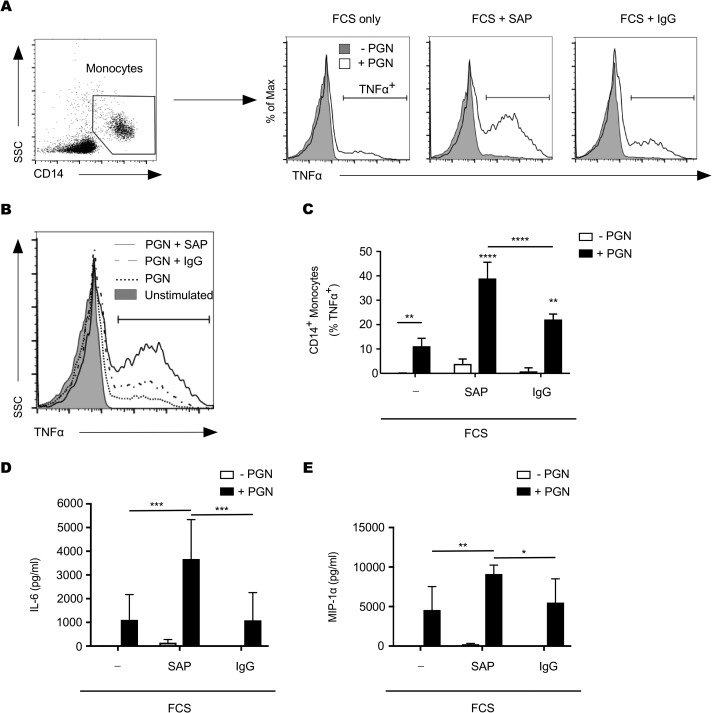
SAP supports better monocyte TNF-α responses than IgG. PBMCs were stimulated for 12 h with PGN in the presence of 1% FCS alone or 1% FCS supplemented with either recombinant SAP (40 μg/ml) or IgG (200 μg/ml). (A) Representative plots and gating strategy used to identify TNF-α-producing CD14^+^ monocytes. (B) Representative histogram overlay comparing PGN-induced TNF-α fluorescence in CD14^+^ monocytes in the presence of FCS, FCS supplemented with IgG, and FCS supplemented with recombinant SAP. (C). TNF-α graph represents means ± SD from 4 individual donors. (D) Multiplex analysis of IL-6 and MIP-1α concentrations in the supernatant of PBMCs stimulated as described above. Unless connected by a line, significance shown is that compared to cells stimulated with PGN in the presence of FCS only. *P* < 0.05 (*), *P* < 0.01 (**), *P* < 0.001 (***), and *P* < 0.0001 (****) by two-way ANOVA (C) or repeated-measures two-way ANOVA (D and E) with Bonferroni posttest.

To compare the relative abilities of SAP and IgG to support PGN-induced cytokine production, we stimulated paired PBMCs with PGN in the presence of 1% fetal calf serum (FCS) supplemented with either recombinant SAP or IgG for 12 h. As shown in Fig. 3A to C, we were able to detect a significantly greater percentage of TNF-α-positive monocytes stimulated with PGN in the presence of recombinant SAP than unopsonized PGN or IgG-opsonized PGN. Not only did more cells produce TNF-α in the presence of recombinant SAP, but the amount of TNF-α produced appeared greater, as indicated by the fluorescence intensity (Fig. 3B). The percentages of TNF-α-producing monocytes in response to PGN from a number of similar experiments are shown in Fig. 3C. These results indicate that SAP is superior to IgG at supporting PGN-driven TNF-α production by monocytes.

We focused on TNF-α production as a measure of monocyte activation to compare the relative contributions of SAP and IgG. Nevertheless, we previously identified other cytokines and chemokines that were secreted during whole-blood stimulation with PGN (17). Therefore, we investigated the ability of SAP and IgG to support the production of a second representative inflammatory cytokine, interleukin-6 (IL-6), and the monocyte chemokine macrophage inflammatory protein 1α (MIP-1α) by PBMCs in response to PGN. Following PBMC stimulation with PGN for 12 h, we observed a superior increase in both IL-6 and MIP-1α in PBMCs stimulated with PGN in the presence of SAP compared to the level with IgG (Fig. 3D and E). In line with our TNF-α data, these results indicate that SAP is a better supporter of PBMC cytokine and chemokine production in response to PGN than IgG.

### SAP-induced response to PGN requires phagocytosis and involves Syk, Src, and PI3-kinase signaling.

**FIG 4:**
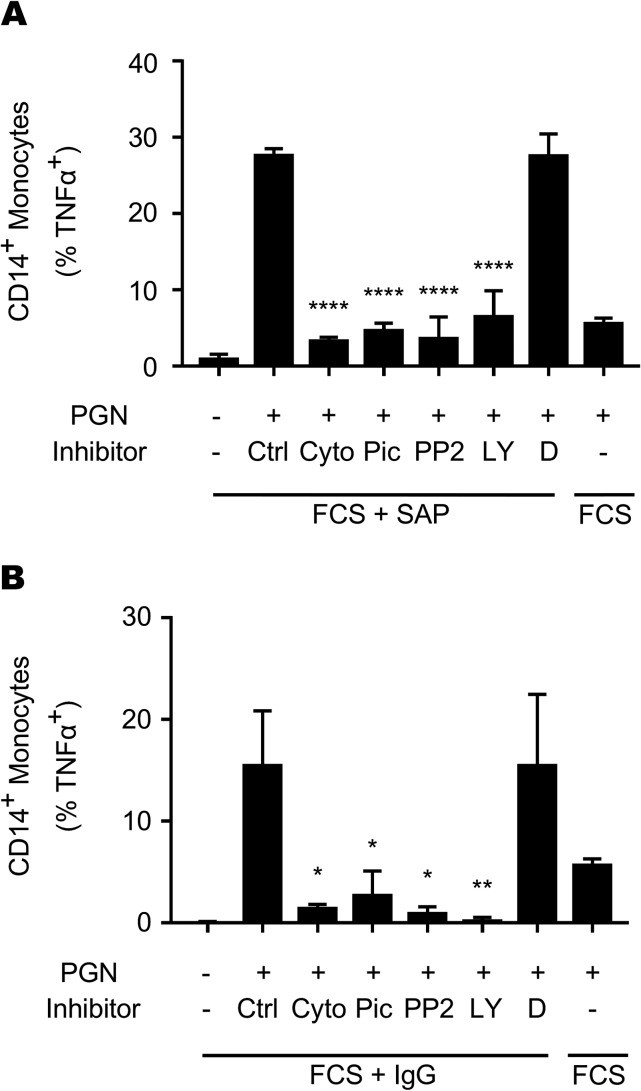
SAP and IgG signaling overlap during PGN stimulation. PBMCs were pretreated for 45 min with cytochalasin D (Cyto; 15 μM), piceatannol (Pic; 50 μM), PP2 (1 μM), LY294002 (LY; 50 μM), or DMSO (D) or were left untreated (Ctrl). Cells were then stimulated for 6 h with PGN in the presence of FCS supplement with or without 40 μg/ml SAP (A) or 200 μg/ml IgG (B). Cells were harvested and stained for CD14 and TNF-α. Data shown represent means ± SD from 2 independent donors. *, *P* < 0.05; **, *P* < 0.01; ****, *P* < 0.0001. Significance was determined by ANOVA with Bonferroni posttest.

Our earlier data showed that PGN-IgG immune complexes required Fc receptor-mediated internalization and lysosomal digestion to elicit a monocyte response. Like IgG, SAP has been shown to bind both human and mouse Fcγ receptors (11, 13). We therefore hypothesized that, like IgG, SAP-mediated monocyte responses to PGN would be dependent on phagocytosis and involve FcγR-dependent signaling pathways. A signaling cascade involving Src family kinases, Syk kinase, and phosphatidylinositol 3-kinase (PI3-kinase) promotes the cytoskeletal rearrangements necessary for Fcγ receptor-mediated phagocytosis (18). To assess the role of signaling mediator involvement in SAP-mediated monocyte responses to PGN, we pretreated PBMCs with the Syk inhibitor piceatannol, the Src inhibitor PP2, the PI3-kinase inhibitor LY294002, or cytochalasin D, a phagocytosis inhibitor. Cells were stimulated for 6 h with PGN in medium containing FCS alone or supplemented with SAP or IgG. We observed that SAP-supported TNF-α production was significantly blocked by inhibition of all three kinases (Fig. 4A). These results were similar to those for IgG-supported responses (Fig. 4B), known to use Src, Syk, and PI3-kinase (18). These data are consistent with the notion that SAP and IgG rely on a similar FcγR signaling cascade to support monocyte responses to PGN, although the results do not establish FcγR involvement.

### SAP-induced PGN responses are independent of complement.

**FIG 5:**
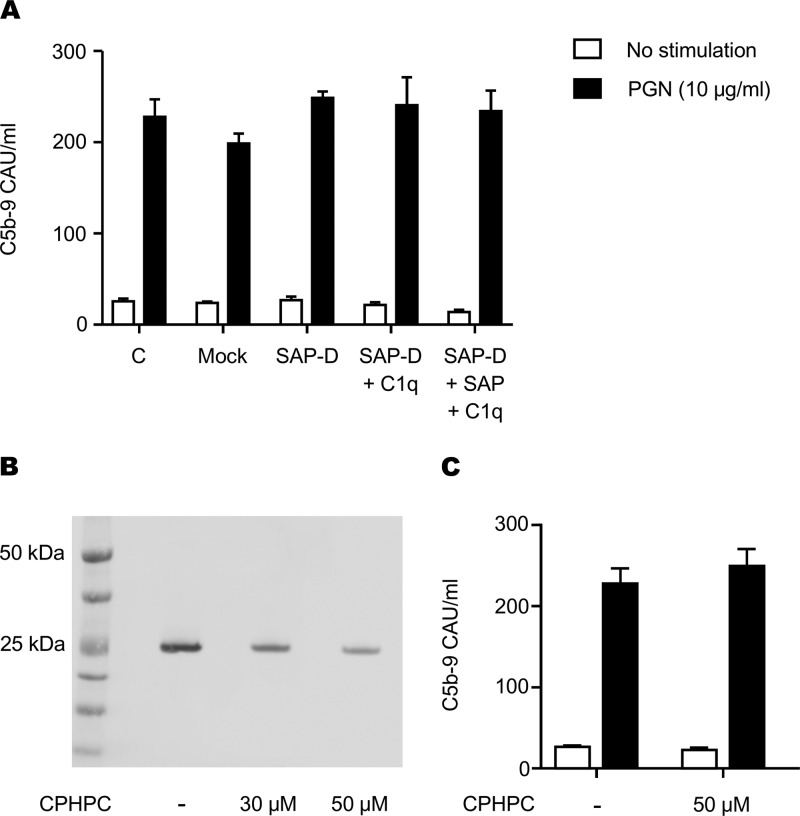
SAP does not influence PGN-induced formation of the terminal complement complex. (A) Pooled serum from three donors was left unmanipulated (C), mock depleted (Mock), or depleted of serum amyloid P (SAP-D). C1q-normalized SAP-depleted serum was reconstituted with or without recombinant SAP and incubated with PGN for 30 min at 37°C. Formation of the terminal complement complex was assayed by ELISA. (B) Serum SAP was inhibited with increasing amounts of CPHPC for 30 min at room temperature prior to PGN interaction. SAP pulldown was performed as described for Fig. 2. (C) Serum SAP was inhibited with 50 μM CPHPC for 30 min before PGN-dependent complement activation. Formation of the terminal complement complex was assessed by ELISA as described for panel A. Data shown are representative of 3 independent experiments.

Our earlier data showed that PGN was able to activate the complement cascade after IgG opsonization (19). Likewise, SAP was shown to interact with components of the complement pathway (15, 16). We therefore tested whether SAP, like IgG, can support PGN-induced activation of the complement cascade and formation of the C5b-9 terminal complement complex (TCC). For these studies, we generated SAP-depleted serum using DNA-cellulose and found that the depletion process resulted in the partial depletion of C1q (data not shown). We therefore tested SAP-depleted or mock-depleted sera that were not reconstituted or were reconstituted with C1q to its normal level found in circulation (60 μg/ml). The depleted serum was then incubated with PGN, and complement activation progressing to TCC was determined using our previously established ELISA protocol (19). We found that SAP-depleted serum was able to support PGN-triggered complement activation as well as the control serum, regardless of the depleted C1q (Fig. 5A). Adding back recombinant SAP or C1q did not change the result. We also used R-1-(6-(R-2-carboxypyrrolidin-1-yl)-6-oxohexanoyl)pyrrolidine-2-carboxylic acid (CPHPC), an inhibitor of SAP (20), to test the effect of SAP inhibition on complement activation in the presence of all serum constituents. SAP binding assays conducted as described for Fig. 2 show that CPHPC reduces SAP binding to PGN in a dose-dependent manner (Fig. 5B). We then measured TCC formation in serum in the presence or absence of 50 μM CPHPC. We found that CPHPC had no effect on PGN-induced TCC formation (Fig. 5C) despite robustly inhibiting SAP-PGN interaction. These findings show that although SAP binds PGN, it cannot support activation of complement, unlike IgG (19).

## DISCUSSION

PGN opsonization by serum factors is important in initiating immune responses to this bacterial PAMP. Here, we investigated the relative abilities of SAP and of IgG to promote innate cellular and humoral immune responses to the DAP-type PGN of B. anthracis. Our findings reported here indicate that SAP functioned as a serum opsonin of DAP-type PGN and that SAP was superior to IgG in promoting cellular proinflammatory responses in monocytes. However, unlike IgG, SAP failed to support PGN-induced complement activation and formation of the biolytic TCC. These findings add to the growing body of evidence that SAP can contribute to the response of the host to systemic infection.

Previous findings showed that SAP could bind to and opsonize the Lys-type S. aureus-derived PGN. We utilized *in vitro* assays to evaluate the ability of SAP to bind to the DAP-type B. anthracis-derived PGN and to support monocyte activation by PGN. The response supported by SAP was sensitive to cytochalasin D treatment, thus showing a requirement for phagocytosis. Phagocytosis is also required for IgG-supported PGN responses (7–9). This is in agreement with a model whereby PGN requires internalization and lysosomal digestion for a response by innate immune cells via NOD1/2 recognition (7).

Our earlier studies on IgG as a PGN opsonin showed that human Fcγ receptors were able to participate in PGN uptake (9, 19). We used Fcγ receptor-blocking antibodies to test the involvement of Fcγ receptors in recognizing SAP-opsonized PGN. In our hands, the blocking antibodies we tested did not prevent SAP-opsonized PGN uptake by monocytes despite blocking IgG-mediated events. Similar results have been observed in studies investigating SAP-mediated phagocytosis and cellular adherence (21). SAP- and IgG-opsonized particles engage the Fcγ receptors at somewhat different sites (12). Specific sites at which commercial Fc blocking antibodies bind Fcγ receptors are not clear, but it is likely that the antibodies were screened based on IgG blocking and not SAP blocking, possibly explaining their failure to prevent SAP-mediated internalization. Pharmacologic inhibition of kinases known to be required for Fcγ receptor signaling shows that both IgG- and SAP-opsonized PGN are sensitive to the same set of kinase inhibitors (Fig. 4). While these kinases are not exclusively involved in FcγR signaling, the data are consistent with FcγR involvement by SAP-opsonized PGN. Further studies in cellular models expressing defined receptors as opposed to primary cells used here are required to establish or eliminate FcγR-mediated internalization.

We found that SAP, in the absence of other human serum constituents, is better than IgG at supporting monocyte activation in response to PGN, as determined by TNF-α production. Furthermore, our multiplex analysis of IL-6 and MIP-1α indicates that SAP is superior at supporting PBMC secretion of other proinflammatory proteins, at least *in vitro*. The superior ability of SAP over IgG in supporting PGN responses may be due to the carbohydrate component of the PGN molecule. IgG shows a modest affinity for carbohydrate antigens (22), while SAP exhibits high affinity for carbohydrates such as lipopolysaccharide (LPS) and PGN (10, 23). It may be that in serum, SAP displaces weaker-affinity immunoglobulins from this PAMP to better promote cellular innate responses.

Complement is a critical component of innate immune responses to pathogens in the blood and is known to play an important role in sepsis pathophysiology (24–26). We showed that PGN primarily triggers the classical pathway of complement activation with additional input from the lectin-dependent pathway (19). However, pentraxin-mediated formation of the terminal complement complex C5b-9 was reported to be inefficient relative to IgG (27). Here, we investigated the formation of TCC as a measure of SAP-opsonized PGN to promote complement cascade activation and progression. We found that, unlike IgG (19), neither depletion nor inhibition of SAP had any effect on PGN-induced TCC formation, indicating that SAP does not support formation of the TCC. Nevertheless, these data do not exclude SAP interaction with upstream complement components to enhance opsonization of PGN. In fact, a recent report shows that SAP interacts with multiple upstream components of the complement cascade (28). Consistent with the latter study, we found that depletion of SAP also led to partial depletion of C1q. It is possible that SAP acts as an immunomodulatory molecule to drive innate cellular responses at the cost of limiting complement-mediated events, although this requires further investigation.

These findings have led us to refine our previous model in which IgG interacts through Fcγ receptors and complement activation to induce a proinflammatory state in monocytes. We now add SAP to this model as an opsonin that works preferentially to modulate PGN-induced inflammation through phagocytosis and subsequent intracellular processing to drive the activation of monocytes. While IgG supports the activation of complement in response to PGN, SAP does not support the formation of terminal complement complexes. However, the interplay between SAP and IgG immune functions has yet to be elucidated in an *in vivo* model, and this elucidation is required to understand the contribution of SAP to sepsis pathophysiology induced by PGN.

## MATERIALS AND METHODS

### Materials.

Allophycocyanin (APC)-conjugated mouse anti-human TNF-α (clone MAb11), APC-conjugated mouse IgG1 isotype control (clone P3.6.2.8.1), phycoerythrin (PE)-Cy7-conjugated mouse anti-human CD14 (clone 61D3), PE-Cy7-conjugated mouse IgG1 isotype control (clone P3.6.2.8.1), and brefeldin A were purchased from eBioscience (San Diego, CA). Rabbit monoclonal anti-human SAP (clone EP1018Y) and rabbit monoclonal anti-human IgG (clone EPR4421) were purchased from Abcam (Cambridge, MA). Recombinant human SAP was purchased from BioLegend (San Diego, CA). Human intravenous immunoglobulin G (IgG) was used as a source of purified human IgG and was kindly provided by Grifols Therapeutics (Los Angeles, CA). Ultralow IgG fetal calf serum (FCS) was purchased from Gibco (Waltham, MA). Single-stranded DNA cellulose was acquired from Sigma-Aldrich (St. Louis, MO). A custom Milliplex human cytokine/chemokine magnetic bead kit was purchased from Millipore Sigma (Burlington, MA).

### Preparation of Bacillus anthracis PGN.

Bacillus anthracis Delta Sterne PGN was prepared using nonpyrogenic labware and endotoxin-free water for all solutions as previously described (8, 9). Briefly, four 500-ml cultures of vegetative bacteria were collected after overnight growth in Trypticase soy broth and subsequently washed once with endotoxin-free water. The cultures were collected by centrifugation at 15,000 × *g* for 10 min at 4°C and then resuspended and boiled three times in 8% UltraPure SDS (Invitrogen). After the final boil, the material was treated twice with DNase/RNase for 30 min at room temperature, followed by washes with endotoxin-free water and centrifugation as described above. Hydrofluoric acid was added to the material overnight at 4°C in order to remove any teichoic acid residues. Following hydrofluoric acid treatment, the material was washed and resuspended in proteinase K solution overnight at 50°C. Proteinase K digestion was conducted twice, followed by washing and subsequent sonication. The now-purified PGN was then boiled in 8% SDS, washed, and dried. PGN was weighed and resuspended in endotoxin-free water at 20 mg/ml.

### Anti-PGN titer assay.

ELISA plates were coated at room temperature overnight with 200 μg/ml PGN in carbonate buffer, pH 9.6. Following the overnight incubation, plates were washed and blocked with 4% bovine serum albumin (BSA) in phosphate-buffered saline (PBS). Serum from human donors was diluted in blocking buffer and incubated with PGN-coated wells for 1 h at room temperature. Plates were washed and incubated with 0.5 mg/ml horseradish peroxidase-conjugated mouse anti-human IgG (BD Biosciences) diluted in 1% BSA–PBS for 1 h at room temperature. The plates were washed and developed with 100 μl/well chromogenic *o*-phenylenediamine (Sigma-Aldrich) solution for 5 to 20 min followed by acid stop. Absorbance was read at 492 nm with wavelength correction at 650 nm.

### Pulldown assay.

Serum without pretreatment or pretreated with 2.5 mM EGTA was incubated with PGN in the presence of a protease and phosphatase inhibitor cocktail (Sigma-Aldrich) for 1 h at 4°C. The opsonized PGN was collected by centrifugation at 20,000 × *g* for 5 min at 4°C. The PGN was washed 3 times with PBS and recovered by centrifugation. PGN was resuspended in NuPAGE LDS sample buffer under reducing conditions (Life Technologies), and bound proteins were eluted by heating for 10 min at 70°C. The eluted proteins and PGN were separated by centrifugation as described above, and the supernatant was saved for analysis.

### PAGE and Western blotting.

Equal volumes of eluates from control and experimental conditions were run on NuPAGE Bis-Tris gels according to the manufacturer's protocol (Life Technologies). For Western blotting, separated proteins were transferred to polyvinylidene difluoride membranes in NuPAGE transfer buffer containing 20% (vol/vol) methanol according to the manufacturer's instructions (Life Technologies). Membranes were washed, blocked for 1 h with 5% (wt/vol) dry milk in Tris-buffered saline containing 0.1% Tween 20, washed again, and incubated with primary antibodies overnight at 4°C. Following overnight incubation, membranes were washed, incubated with secondary antibodies for 1 h at room temperature, washed again, and imaged on the Odyssey Imager (LI-COR Biotechnology). Membranes were stripped and reprobed when necessary.

### PBMC isolation and culture.

Peripheral blood from healthy donors was collected by venipuncture into heparinized vacutainers according to a protocol approved by the Oklahoma Medical Research Foundation Internal Review Board. PBMCs were isolated via density gradient centrifugation using Histopaque-1077 (Sigma-Aldrich) according to the manufacturer's protocol. PBMCs were washed and reconstituted in RPMI containing FCS (1% vol/vol) and GlutaMAX-1 (2 mM). The cells were cultured in the presence of recombinant SAP, IgG, or whole human serum. Cells were stimulated as described in Results. Brefeldin A (3.0 μg/ml) was added to inhibit cytokine secretion, and polymyxin B (10 μg/ml) was used to inhibit any LPS contamination. In some experiments, cytochalasin D (15 μM), piceatannol (50 μM), PP2 (1 μM), or LY294002 (50 μM) was added 45 min prior to PGN stimulation and included throughout the experiment.

### Flow-cytometric analysis.

TNF-α production in monocytes was assessed by stimulating 4.5 × 10^5^ to 9.0 × 10^5^ PBMCs with PGN (10 μg/ml) in 100 μl for 6 or 12 h at 37°C in the presence of 1% FCS supplemented with recombinant SAP, IgG, or human serum. Cells were then washed in PBS and blocked with 10% human AB serum (Sigma-Aldrich) in PBS. Cells were fixed, permeabilized, and stained with anti-CD14 and anti-TNF-α antibodies. Stained cells were then analyzed on a FACSCelesta flow cytometer (BD Biosciences), where a minimum of 5,000 events identified by forward and side scatter as monocytes were collected per sample. Paired isotype and unstained controls were used to set up the gating thresholds for each antibody.

### Multiplex analysis.

The production of IL-6 and MIP-1α was assessed by stimulating 4.5 × 10^5^ to 9.0 × 10^5^ PBMCs with PGN (10 μg/ml) in 100 μl for 12 h at 37°C in the presence of 1% FCS supplemented with recombinant SAP, IgG, or human serum. The cultures were collected and centrifuged at 500 × *g* to remove cells. Supernatants were harvested and stored at −80°C until they could be processed according to the manufacturer's protocol (Millipore Sigma). Processed samples were then analyzed using a BioPlex 200 (Bio-Rad).

### Depletion of SAP from human serum.

SAP was depleted from fresh serum as previously described (14). Briefly, fresh human serum was incubated twice with DNA cellulose on ice for 1-h intervals. After incubation, the depleted serum was sterile filtered and stored at −80°C.

### Terminal complement complex assay.

Pooled SAP-depleted serum or whole serum was diluted once with GVB++ reagent buffer containing 0.1% gelatin, 5 mM barbital (Veronal), 145 mM NaCl, 0.025% NaN_3_, and 2 mM CaCl_2_. In some experiments, depleted serum was reconstituted with recombinant SAP (40 μg/ml), or SAP was inhibited with 50 μM CPHPC (Cayman Chemical). Complement activation was initiated with PGN (10 μg/ml). Samples were incubated for 30 min at 37°C, and then reactions were stopped with 10 mM EDTA. Terminal complement complex formation was assessed using an ELISA method previously described (19).

### Statistical analysis.

Statistical analyses were conducted using GraphPad Prism software. For all studies, means between groups were analyzed using a one-way analysis of variance (ANOVA), a two-way ANOVA, or a repeated-measures two-way ANOVA with a Bonferroni posttest. A *P* value of ≤0.05 was considered significant.
